# Examination of Bacterial Inhibition Using a Catalytic DNA

**DOI:** 10.1371/journal.pone.0115640

**Published:** 2014-12-22

**Authors:** Long Qu, M. Monsur Ali, Sergio D. Aguirre, Hongxia Liu, Yuyang Jiang, Yingfu Li

**Affiliations:** 1 Department of Chemistry, Tsinghua University, Beijing, China; 2 Department of Biochemistry and Biomedical Sciences & Michael G. DeGroote Institute for Infectious Disease Research, McMaster University, Hamilton, Ontario, Canada; 3 The State Key Laboratory Breeding Base & Shenzhen Key Laboratory of Chemical Biology, Graduate School at Shenzhen, Tsinghua University, Shenzhen, China; Florida International University, United States of America

## Abstract

Determination of accurate dosage of existing antibiotics and discovery of new antimicrobials or probiotics entail simple but effective methods that can conveniently track bacteria growth and inhibition. Here we explore the application of a previously reported fluorogenic *E. coli*-specific DNAzyme (catalytic DNA), RFD-EC1, as a molecular probe for monitoring bacterial inhibition exerted by antibiotics and for studying bacterial competition as a result of cohabitation. Because the DNAzyme method provides a convenient way to monitor the growth of *E. coli*, it is capable of determining the minimal inhibitory concentration (MIC) of antibiotics much faster than the conventional optical density (OD) method. In addition, since the target for RFD-EC1 is an extracellular protein molecule from *E. coli*, RFD-EC1 is able to identify pore-forming antibiotics or compounds that can cause membrane leakage. Finally, RFD-EC1 can be used to analyse the competition of cohabitating bacteria, specifically the inhibition of growth of *E. coli* by *Bacillus subtilis*. The current work represents the first exploration of a catalytic DNA for microbiological applications and showcases the utility of bacteria-sensing fluorogenic DNAzymes as simple molecular probes to facilitate antibiotic and probiotic research.

## Introduction

Antibiotic resistance represents a serious global crisis and is an inevitable result of antibiotic use [1]. Excessive antibiotic usage works as a selection pressure that drives the emergence of antibiotic-resistant strains of bacteria [2]–[4]. Sensible use of antibiotics has now been widely recognized as an important strategy to alleviate the issue of antibiotic resistance and the accepted practices to implement this strategy include the use of adequate dosage, proper selection of initial empiric regimens (i.e. type of antibiotic and its dosage), and identification of resistant bacterial strains [4]–[6]. However, antibiotic resistance is an inevitable on-going evolutionary process, and thus, searching for novel antibiotics or alternative treatment methods constitutes never-ending efforts in research and development. Searching Nature's large repertoire of small molecules for antibiotics has proven to be highly fruitful [7], [8]. Discovering antimicrobial peptides (AMPs) represents a growing interest in antibiotic discovery largely because AMPs are broadly effective and operate by membrane-targeting pore-forming mechanism, and thus it is difficult for bacteria to develop resistance [9]–[12]. Exploring benign bacteria to contain pathogens, which serves as a probiotic strategy to restore balanced microbiota, represents a highly promising alternative strategy for fighting bacterial infections, particularly those caused by antibiotic-resistant strains of bacteria [13]–[18]. For example, *Bacillus subtilis* was proved to be potential probiotics in poultry [19], [20], pigs [21] and aquaculture [22].

Both determination of accurate dosage of existing antibiotics and discovery of new antimicrobials or probiotics need effective methods that can facilely report bacterial inhibition. Measuring cell concentrations by optical density (OD) and counting cells using agar plating are the two widely used methods for this purpose [23], [24]. Although both methods are relatively easy to use, they require a fairly long cell-growing step and thus are time-consuming processes. In addition, growing pathogens to a high cell density that is inherent to these methods also represents a serious biosafety concern. Modern molecular methods, such as amplifying the genomic DNA of a pathogen by polymerase chain reaction (PCR) or detecting a specific bacterial antigen using an antibody, are more sensitive and rapid. However, they are not ideal for antibiotic discovery (which is often carried out in high-throughput fashion) due to the requirement of multiple steps and expensive reagents [25]–[28]. For these considerations, there is a significant demand for simple but effective bacteria-tracking methods. For example, the pore-forming activity of AMPs is currently assessed using artificial membrane [29]–[33], which is not compatible with high-throughput screening (HTS). A convenient assay that works with live bacterial cells and compatible with HTS would be more desirable. Furthermore, examination of bacterial competition can not only lead to the discovery of inhibitory molecules [18], [34] and probiotics [35], [36] for pathogens, but is also important for quorum-sensing [37] and analysis of resource ratio model of competition (a theory for predicting relationship between abilities of species to use resources, resource usability and the result of competitive interactions) [38], [39]. The bacterial competition is currently observed using a rather tedious procedure: sample aliquots are first taken at chosen intervals; the bacteria in the mixture are either inoculated onto specific agar plates, cultured or counted [40], [41] or identified using multi-step immunoassays [42], [43]. Both methods are neither convenient for discovering potential probiotics for a given pathogen from a large collection of candidate bacteria, nor for testing probiotic activity of common bacteria against different pathogens. Simpler methods that can avoid such a lengthy procedure would be necessary.

**Figure 1:**
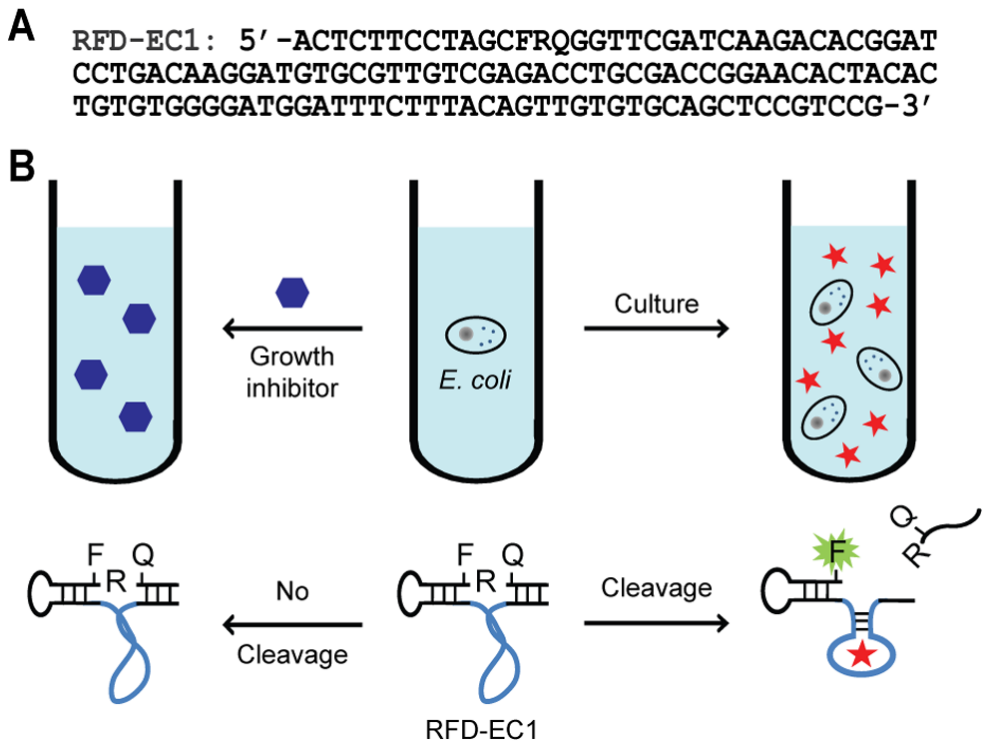
Utilization of a bacterium-sensing DNAzyme for the study of bacterial inhibition. (A) The sequence of RFD-EC1. All nucleotides in the sequence are deoxyribonucleotides except for F, Q and R, which denote fluorescein-dT, dabcyl-dT, adenine ribonucleotide, respectively. (B) Conceptual framework for utilizing RFD-EC1 to facilitate the study of bacterial inhibition. Growth inhibitors: antibiotics or probiotics. The presence of an inhibitor prevents the growth of *E. coli* cells, which will not produce the activator (star) to induce the cleavage of RFD-EC1.

We have been interested in developing RNA-cleaving fluorogenic DNAzymes (RFDs) as simple bacterial indicators [44]–[49]. Such DNAzymes are evolved to cleave, in the presence of a specific bacterium, a DNA substrate that contains a single RNA unit as the cleavage site, which is sandwiched between two nucleotides modified with a matching pair of fluorophore and quencher. Such setup links fluorescence signal production to bacteria-activated DNAzyme activity without the need for a separation step. For this reason, RFDs are excellent molecular probes for bacterial detection. We have previously reported an *E. coli*-sensing RFD, named RFD-EC1 (its sequence is provided in Fig. 1A), that has some interesting properties [50]–[53]. This probe not only exhibits extremely high recognition specificity for *E. coli*, it also responds to a protein target that exists extracellularly [50], [54]. Because of these two traits, in this study we set out to explore the application of RFD-EC1 as a unique molecular probe for monitoring bacterial inhibition exerted by antibiotics and for studying bacterial competition as a result of cohabitation (schematically illustrated in Fig. 1B).

## Results and Discussion

### Comparison of *E. coli* growth curves via optical density reading and the cleavage of RFD-EC1

**Figure 2:**
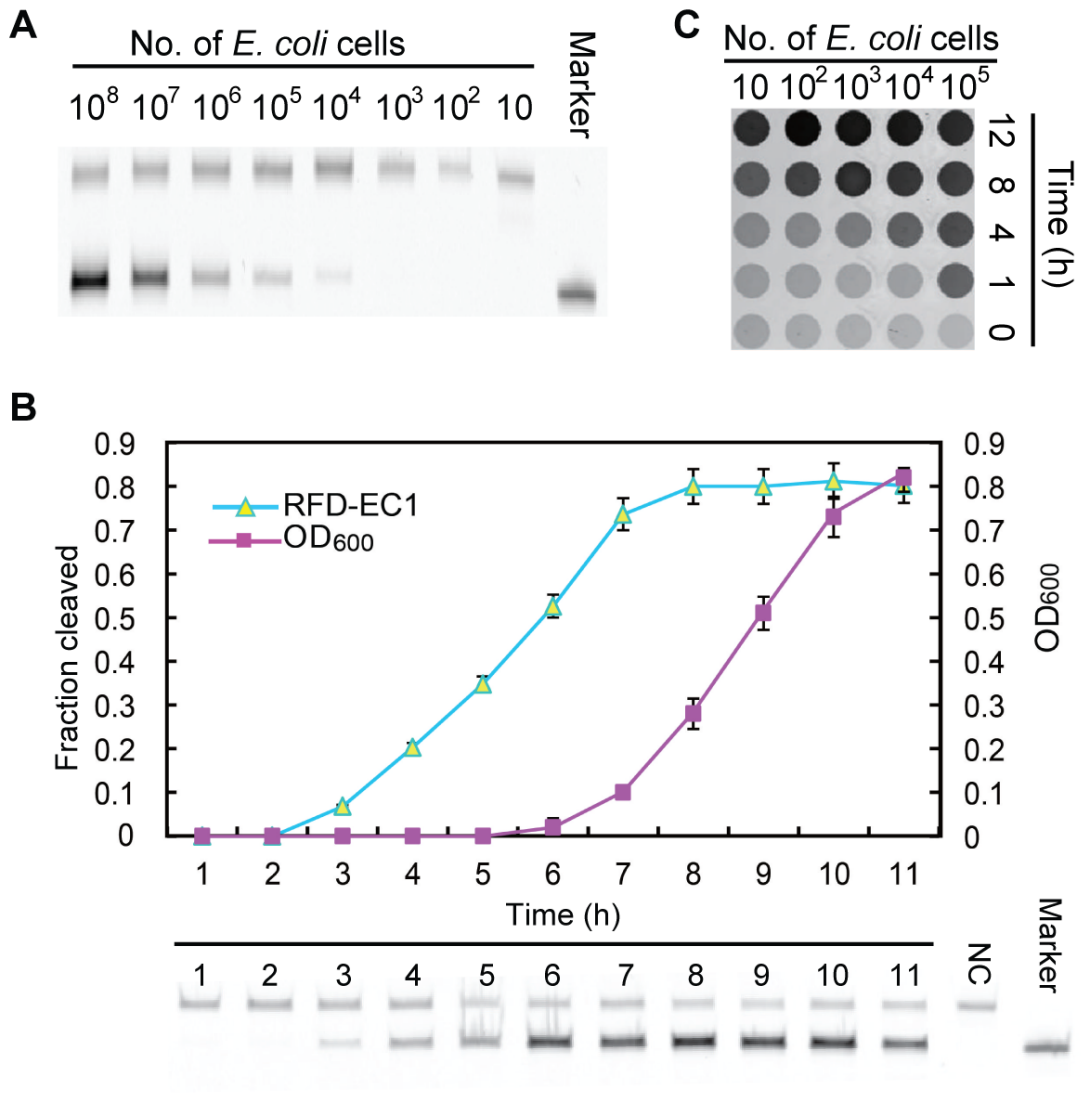
*E. coli* growth curves. (A) The detection limit of RFD-EC1 for *E. coli* measured by dPAGE analysis of 2 pmol RFD-EC1 following 1-h incubation in 50 µL of reaction buffer (1× RB) containing 10, 100, 10^3^, 10^4^, 10^5^, 10^6^, 10^7^ or 10^8^ *E. coli* cells. (B) *E. coli* growth curves measured by RFD-EC1 and optical density method with 100 seeding cells. NC  =  negative control (no *E. coli*), Marker  =  RFD-EC1 treated in 2 M NaOH at 90°C for 10 min. (C) Tracking *E. coli* cells growth by RFD-EC1 in microwell plate. The culture time was 0, 1, 4, 8, 12 hours, and the number of seeding cells was 10, 10^2^, 10^3^, 10^4^ and 10^5^ respectively. 50 µL from each 200-µL culture was used for the cleavage reactions.

RFD-EC1 cleaves a fluorogenic chimeric DNA/RNA substrate that contains a single ribonucleotide (as the cleavage site) located between a fluorescein-modified deoxythymidine and a dabcyl (the quencher of fluorescein)-modified deoxythymidine (Fig. 1A) [42]–[46]. We first examined the detection limit of RFD-EC1 for *E. coli* through the analysis of cleavage product of RFD-EC1 upon one-hour incubation with varying numbers of cells in 1× reaction buffer (1× RB, which contains 50 mM HEPES, 150 mM NaCl, 15 mM BaCl_2_, 0.01% Tween 20, pH 7.5); the result is shown as Fig. 2A. Using this method, RFD-EC1 afforded a limit of detection of 10^4^ cells, which is consistent with our previous finding [54].

We obtained the cell growth curve through periodical reading of the optical density of an *E. coli* culture solution with 100 seeding cells in 1.5 mL of Luria Bertani (LB) broth. For comparison, we also obtained a curve of RFD-EC1 cleavage as a function of *E. coli* growth time by measuring the fraction cleavage of RFD-EC1 in one hour after it was exposed to 25 µl of the same *E. coli* culture taken at specific time points (in 1× RB). While the RFD-EC1 based method was able to detect the cell growth in 3 hours, the OD method needed 7 hours. Furthermore, the cleavage method reached the plateau (full cleavage) in 7 hours; in contrast, the OD method just began to reach the stationary phase in 11 hours. Thus, the RFD method is much more sensitive than the OD method. We also analysed the detection sensitivities of both methods in terms of the number of cells that can be detected. While the OD method required at least ∼10^6^ cells (S1A Fig.), the RFD-EC1 cleavage method can detect as low as ∼10^4^ cells (S1B Fig.), representing ∼100-fold difference in sensitivity.

We also examined if the RFD-EC1 could be used to follow the growth of *E. coli* in 96-well microplates without conducting PAGE analysis. As shown in Fig. 1C, the growth of varying numbers of *E. coli* cells indeed can be easily tracked using this method: when the number of seeding cells was 10, clearly detectable signal can be achieved following 8 hours of culturing; when the number of seeding cells increased to 10^5^, the culturing time can be dropped to 1 hour. For the sensitivity comparison, the microplate method can detect ∼10^5^ cells (S1C Fig.), which is 10-fold more sensitive than the OD method.

### Determining bacterial inhibition by ampicillin using RFD-EC1

**Figure 3:**
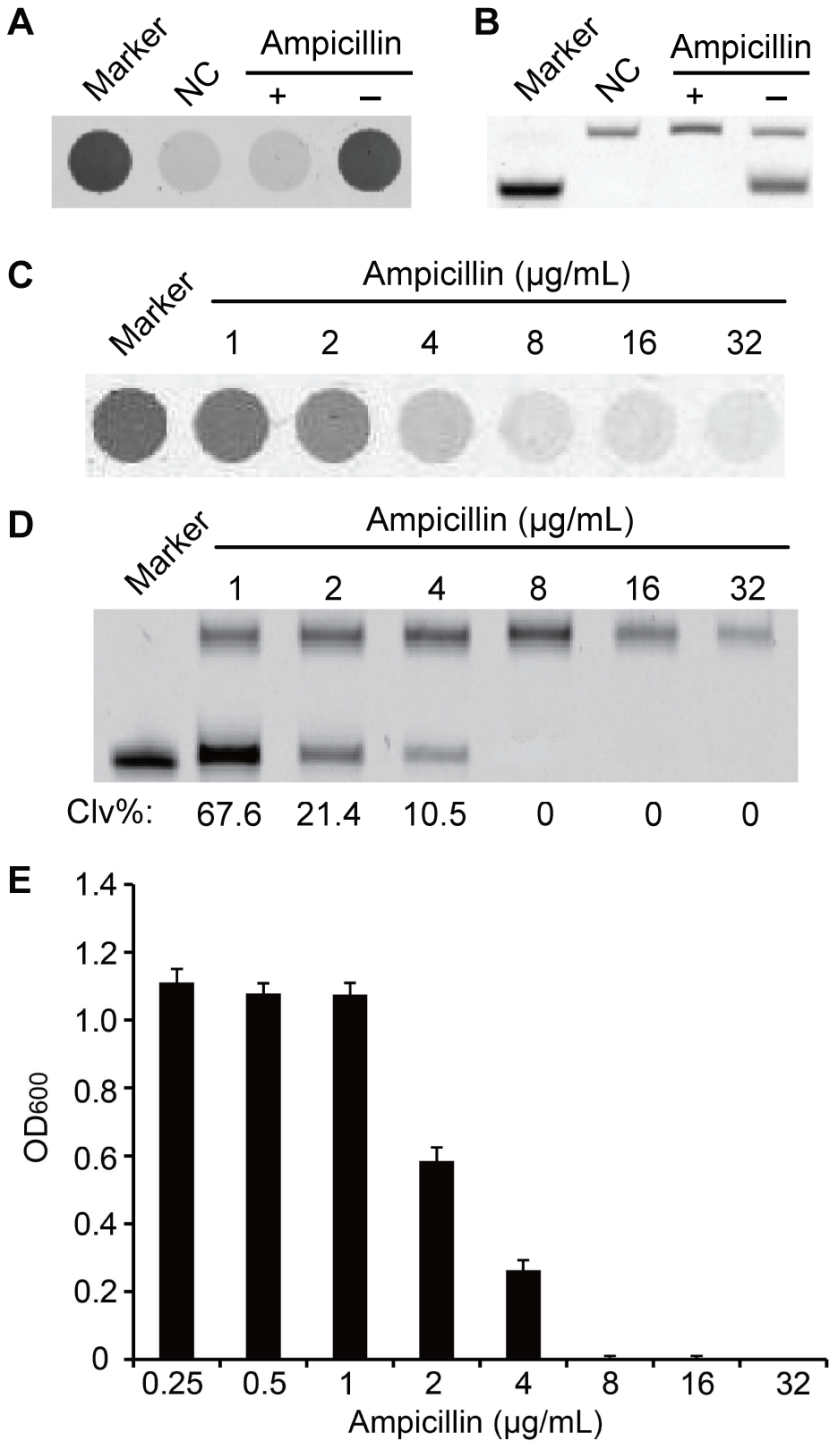
Inhibition of growth of *E. coli* by ampicillin. (A) Microwell plate based signaling profiles of RFD-EC1 in the presence of 100 seeding *E. coli* cells that were incubated at 37°C for 6 hours with or without 10 µg/mL ampicillin in LB. (B) Fluorescent image of 10% dPAGE of the samples in panel A. (C) Growth inhibition test using RFD-EC1 with 100 seeding *E. coli* cells that were incubated at 37°C in LB for 6 hours in the presence of 0, 0.25, 0.5, 1, 2, 4, 8, 16, 32 µg/mL ampicillin. (D) dPAGE analysis of the reaction mixtures in (C). (E) Growth inhibition test using OD method with 2 × 10^5^ *E. coli* cells that were incubated at 37°C in LB for 18 hours in the presence of 0.25, 0.5, 1, 2, 4, 8, 16, 32 µg/mL of ampicillin.

We next examined whether the DNAzyme method was able to determine the optimal amount of antibiotics that can inhibit bacterial growth completely. First, we tested the effect of 10 µg/mL ampicillin, which is known to completely inhibit the growth *E. coli* [55]. A 2-mL LB sample containing 100 seeding *E. coli* cells was incubated at 37°C for 6 hours, followed by a cleavage reaction where 10 µL of the bacterial culture was allowed to react with RFD-EC1 in 1× RB in a well of a 96-well plate (the total volume was 100 µL). As shown in Fig. 3A, although significant fluorescence increase was observed in the bacterial sample that did not have ampicillin, the addition of 10 µg/mL ampicillin led to insignificant fluorescence change. The data in Fig. 3B verifies that the signal increase in the control sample was due to the cleavage of RFD-EC1 and the lack of fluorescence increase was linked to the lack of cleavage of RFD-EC1.

We subsequently investigated if the RFD-EC1 could be used to determine the bacterial growth in relation to the concentration of antibiotics. Six culture tubes containing 2 mL of LB media with a progressively 2-fold diluted series of ampicillin concentrations, from 32 to 1 µg/mL, were prepared. After 6 hours of culture, reactions between RFD-EC1 and each culture were done in a 96-well plate. As shown in Fig. 3C, the fluorescence signal increased in the samples with 1, 2 and 4 µg/mL ampicillin while the samples containing 8, 16, 32 µg/mL ampicillin did not show significant fluorescence enhancement. Fig. 3D reveals that there was no cleavage product found with the samples containing 8, 16, 32 µg/mL ampicillin while increasing amounts of cleavage product were produced when the ampicillin concentrations were decreased to 4, 2 and 1 µg/mL. Thus the MIC of ampicillin obtained with the RFD-EC1 method is 8 µg/mL, which is consistent with the result obtained with the OD method as shown in Fig. 3E. However, the time required to determine the MIC by RFD-EC1 method is only 7 hours whereas OD method needs at least 18 h.

### Assessment of bacterial membrane permeability using RFD-EC1

**Figure 4:**
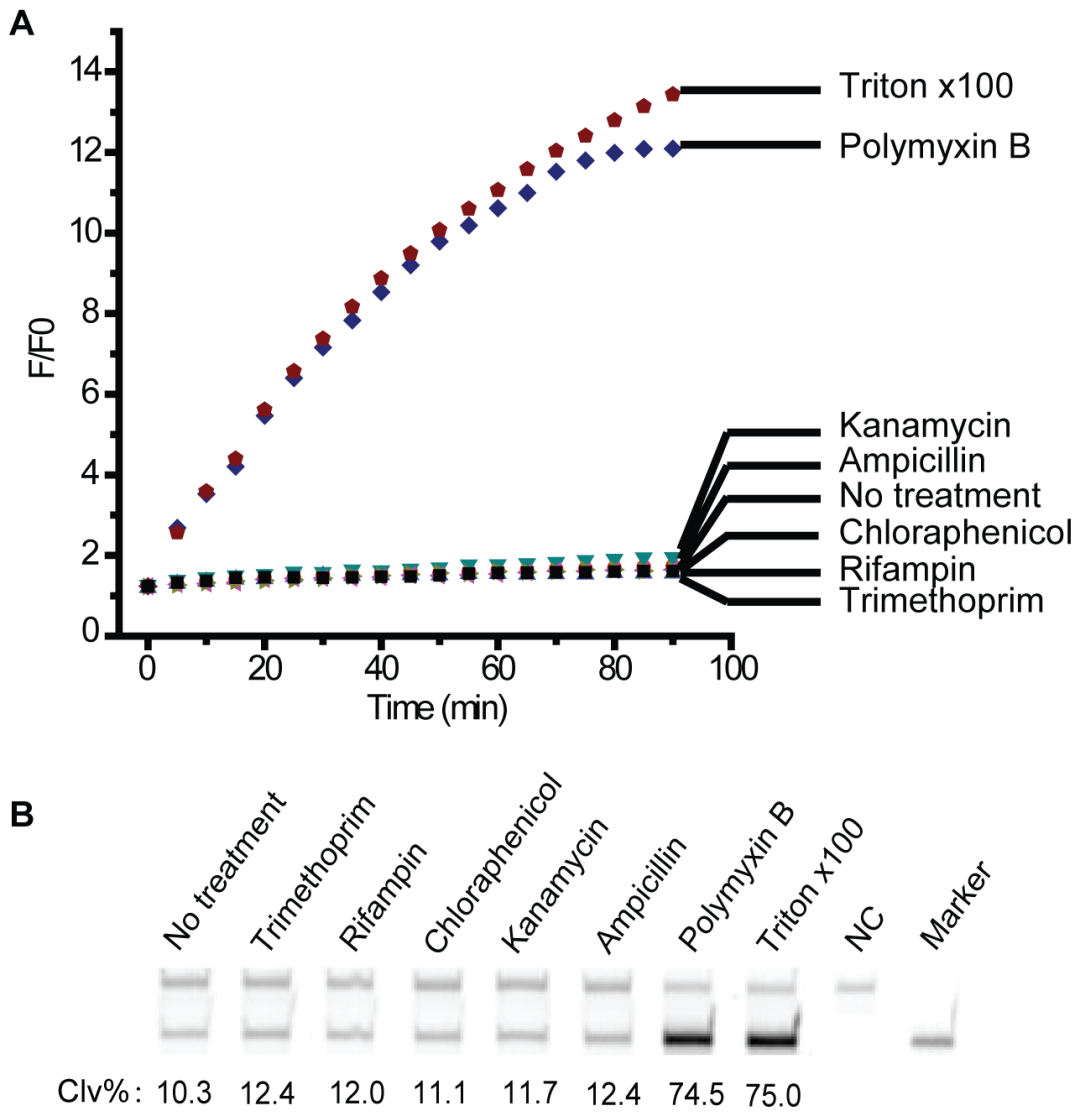
Identification of pore-forming antibiotics using RFD-EC1. (A) The transmembrane pore-forming ability of the chosen antibiotics was assessed by measuring the real time fluorescence of the reaction between RFD-EC1 and 3×10^5^ *E. coli* exposed to 100 µg/mL kanamycin, ampicillin, trimethoprim, chloramphenicol, rifampin, polymyxin B and 0.1% Triton x100 for 15 minutes. (B) dPAGE analysis of the reaction mixtures in (A), the incubation time was 90 min.

RFD-EC1 was isolated to cleave in the presence of the crude extracellular mixture (CEM) of *E. coli* and it has been determined that the target that activates the DNAzyme is a protein molecule based on the observation that the treatment of the CEM with proteases abolishes the DNAzyme activity [17]. Although the identity of this target is yet to be determined, we found that the target protein is much more abundant intracellularly [54]. Based on these observations, we speculate that RFD-EC1 can be used to identify transmembrane pore-forming molecules. Polymyxin B is an antibiotic that is known to bind to the cell membrane, making it more permeable [56], [57]. We examined the cleavage activity of RFD-CE1 in the presence of *E. coli* cells treated with polymyxin, along with five other control antibiotics: kanamycin (which binds ribosomes to inhibit their translocation [58]), ampicillin (inhibiting bacterial cell-wall synthesis [59], [60]), chloramphenicol (which prevents peptidyl reaction [61], [62]), rifampin (inhibiting DNA-dependent RNA synthesis [63]), and trimethoprim (which is an inhibitor of dihydrofolate reductase [64]). We also used Triton X 100 as a positive control, which is known to destroy bacterial cell membrane. Note for this experiment, *E. coli* cells were purified from the CEM by centrifugation – this step serves to remove the protein activator accumulated during cell culturing, which is essential for testing the pore-forming ability of an antibiotic using the DNAzyme. As shown in Fig. 4A, only polymyxin B and Triton X 100 resulted in progressive increase of fluorescence signal. Fig. 4B verifies that the signal increase was due to the cleavage of RFD-EC1.

### Monitoring bacterial cohabitation using RFD-EC1

**Figure 5:**
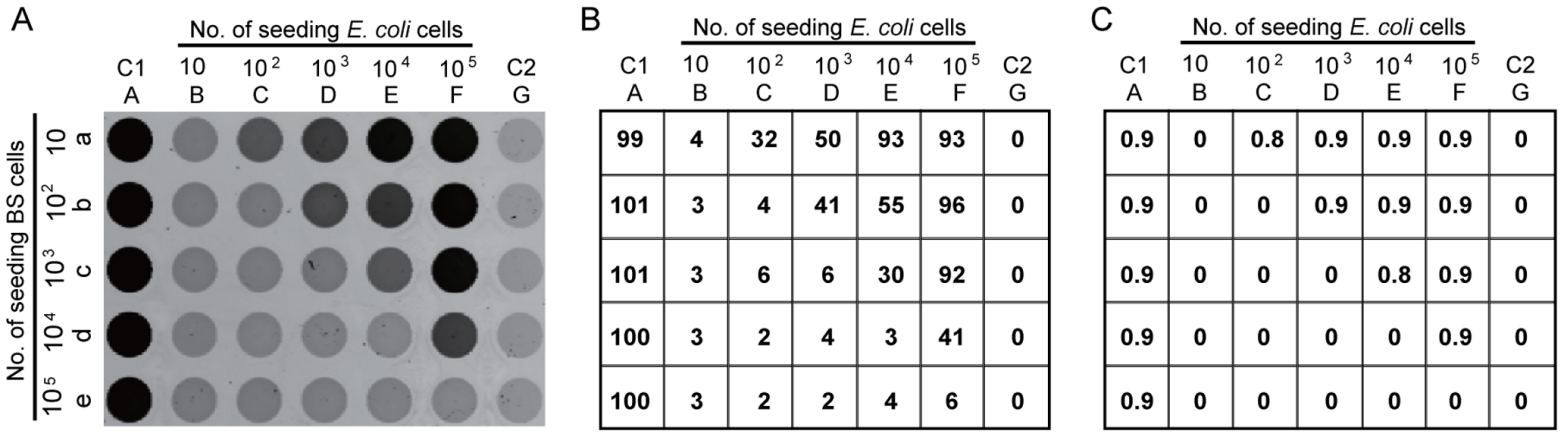
Tracking bacterial competition using RFD-EC1. (A) A microwell plate based assay. Each well contains 10–10^5^ *E. coli* and/or *B. subtilis* cells in 200 µL of LB. Following 18-h incubation at 37°C, 7 µL of the culture from each well was mixed with 93 µL of 1× RB containing 2 pmol RFD-EC1 in a reaction plate. After 1 h, the reaction plate was scanned for fluorescence intensity. (B) Relative fluorescence (RF) of the reaction plate in (A). RF for each well is calculated as: 100× (F_each well_ - F_Ge_)/(F_Aa_ - F_Ge_). (C) Post-competition analysis. Following the competition in (A), each bacterial mixture (10 µL) was added to a well of a new plate with 190 µL of LB containing 50 µg/mL ampicillin. The new plate was incubated at a 37°C for 24 hours to obtain the OD_600_ value.

We next examined the use of RFD-EC1 to study the growth of *E. coli* in the presence of other bacteria. Particularly, we examined the competition of between *E. coli* and *B. subtilis* when they were cultured together (Fig. 5). We designed a 7×5 (column × row) plate assay in which each well was seeded with a defined number of *B. subtilis* and/or *E. coli* cells (Fig. 5A). Specifically, the wells in the column A (C1, Control 1; positive control) represents progressively increased *E. coli* cells starting with 10 cells and ending with 10^5^ cells; the wells in the column G (C2; negative control) had the same arrangement except that *E. coli* cells were substituted with *B. subtilis* cells; each of the remaining wells contained both *E. coli* and *B. subtilis* cells in a combination where the number of *E. coli* cells increased from 10–10^5^ in 10-fold increment from left to right and the number of *B. subtilis* cells increased from 10–10^5^ also in 10-fold increment from top to bottom. The plate was incubated at 37°C for 18 hours to allow the full growth of bacteria; it should be noted that the optical density of each culture well reached ∼0.9 following 18-hour incubation, suggesting that same numbers of cells were produced in each well.

An aliquot of each culture was taken to react with RFD-EC1 in a new plate arranged in the same way. Following the reaction, the assay plate was scanned for fluorescence intensity (Fig. 5A). We then calculated the relative fluorescence of all the wells using the following formulas: (F – F_Ga_)/(F_Ae_ – F_Ga_) where F is the fluorescence reading of a given well, F_Ga_ and F_Ae_ are the readings in the wells of Ae and Ga, representing the theoretically highest and lowest fluorescence readings. Thus derived data is graphically presented in Fig. 5B.

Several observations were made. First, the maximal fluorescence was observed for each well in column A and minimal fluorescence was seen for the wells in column G, which indicates that both positive and negative controls worked well. Second, when the number of seeding *E. coli* cells was equal to or smaller than the number of seeding *B. subtilis* cells, the growth of *E. coli* was completely inhibited, reflected by very small RF values for these wells. Interestingly, there was no detectable signal in the well Fe where 10^5^ *E. coli* cells were seeded along with 10^5^ *B. subtilis* cells. Since we have already shown in Fig. 2C that 10^5^ *E. coli* cells could produce a strong fluorescence signal following as short as 1 hour of culturing, the lack of signal strongly suggests that the seeding *E. coli* cells were either eliminated by *B. subtilis* or the growth of *E. coli* was completely inhibited during 18 hours of culturing so that there was an insufficient amount of the protein target to activate RFD-EC1. Third, the edge of competition of *B. subtilis* over *E. coli*, which is defined here as the fold of excess of *E. coli* cells over *B. subtilis* cells to reach a 50∶50 final population, is 100. In other words, in order for *E. coli* to dominate *B. subtilis* for growth, the seeding *E. coli* cells need to be 100 times over the seeding *B. subtilis* cells. If the ratio of *E. coli* to *B. subtilis* cells drops to 10, *B. subtilis* would partially inhibit *E. coli* growth.

Many *E. coli* and *B. subtilis* combinations in Fig. 5A produced a low level of fluorescence (relative fluorescence between 2–6 on the scale of 100; Fig. 5B), suggesting that the number of *E. coli* cells in these mixtures was too small to activate RFD-EC1. Since these wells were originally seeded with 10–10^5^ *E. coli* cells, we were curious as to whether the *E. coli* cells remained dormant in these wells or the growth of the *B. subtilis* cells has completely wiped out the original *E. coli* cells. Since we used an ampicillin-resistant *E. coli* cell line while the *B. subtilis* cell line that is not resistant to this antibiotic, to address the above question, we carried out a re-culturing experiment in the presence of 50 µg/mL of ampicillin following the cohabitation step. The OD value of each well after re-culturing are shown in Fig. 5C. In the wells with the number of seeding *E. coli* cells is the same as or less than that of seeding *B. subtilis* cells, the *E. coli* cells were vanished. In the wells where seeding *E. coli* cells are 10 times over *B. subtilis* cells, some *E. coli* cells survived the cohabitation. The lack of fluorescence signal in the well Fe where 10^5^ *E. coli* cells were seeded (which should produce a sufficient level of the protein target to activate the DNAzyme) strongly suggest that the protein target from pre-seeded *E. coli* cells were degraded, possibly by proteases from *B. subtilis*.

## Conclusions

We have shown in this study that the *E. coli*-sensing fluorogenic DNAzyme probe RFD-EC1 can be used as a convenient tool for monitoring bacterial inhibition exerted by antibiotics and for studying bacterial competition as a result of cohabitation. For antibiotic testing the DNAzyme method offers a short time to reach the minimal inhibitory concentration of an antibiotic. In addition, due to the fluorogenic nature of the probe, such a method is ideal for assays that need to be conducted in a high throughput manner. Furthermore, the DNAzyme method is able to distinguish membrane-targeting pore-forming antibiotic from other types of antibiotics and thus can be potentially applied for high-throughput screening of pore-forming antibiotics or compounds. For monitoring bacterial competition, the DNAzyme method offers a convenient approach that is impossible with optical density measurement or too cumbersome with culturing-based bacterial composition analysis. Since fluorogenic DNAzymes can be relatively easily isolated from synthetic random-sequence DNA libraries to recognize any given food/water-borne or medically important bacterial pathogen, we expect the described method can find useful applications in future antibiotic and probiotic discoveries.

## Materials and Methods

### Oligonucleotides and Materials

The sequences of all synthetic DNA oligonucleotides are provided in S2 Fig.. The fluorogenic substrate FS1, which contains an adenosine ribonucleotide as the cleavage site flanked by a fluorescein-dT and a dabcyl-dT, was purchased from Yale University Keck Facilities. The other DNA oligonucleotides were purchased from Integrated DNA Technologies (IDT). All oligonucleotides were purified by 10% denaturing (8 M urea) polyacrylamide gel electrophoresis (dPAGE) and their concentrations were determined based on the UV absorbance at 260 nm (Genesys UV 10, Thermo Scientific). T4 DNA ligase, T4 polynucleotide kinase (PNK) and ATP were obtained from Thermo Scientific. All the antibiotics (kanamycin, ampicillin, trimethoprim, chloraphenicol, tetracycline and polymyxin B) and other chemicals were purchased from Sigma-Aldrich. The bacteria *E. coli K12 MG1655* and *Bacillus subtilis 168* are routinely maintained in our laboratory. *Listeria monocytogenesis and Staphylococcus aureus* were obtained from the Shenzhen Centre for Disease Control and Prevention (Shenzhen, Guangdong province, China). Water used here was double-deionized (ddH_2_O) and autoclaved.

### Buffer Conditions

Cleavage reactions of RFD-EC1 were performed in the following 1× reaction buffer (1× RB): 50 mM HEPES (pH 7.5), 150 mM NaCl, 15 mM BaCl_2_ and 0.01% Tween 20.

### Preparation of RFD-EC1

RFD-EC1 was produced through the ligation of FS1 (5′-ACTCT TCCTA GCFRQ GGTTC GATCA AGA-3′; F: fluorescein-dT; Q: dabcyl-dT; R: ribo-A) and EC1 (5′-CACGG ATCCT GACAA GGATG TGTGC GTTGT CGAGA CCTGC GACCG GAACA CTACA CTGTG TGGGA TGGAT TTCTT TACAG TTGTG TGCAG CTCCG TCCG-3′) using LT1 (5′-CTAGG AAGAG TCGGA CGGAG CTG-3′) as the template. 100 µM stock solutions of FS1, EC1and LT1 were first prepared using ddH_2_O. 50 µL of the FS1 stock were then taken for DNA phosphorylation (reaction volume: 100 µL) with 2 µL of 100 mM ATP and 20 units of PNK for 30 min at 37°C in 1×PNK buffer A. The reaction was quenched by heating the mixture at 90°C for 5 min. Equimolar EC1 and LT1 were then added to this solution, and the mixture was heated at 90°C for 40 s and cooled to room temperature for 10 min. Then, 40 µL of 10× T4 DNA ligase buffer was added, followed by the addition of 20 units of T4 DNA ligase. The volume was adjusted to 400 µL with ddH_2_O and the mixture was incubated at room temperature for 2 h. The DNA molecules in the mixture were concentrated by ethanol precipitation and the ligated RFD-EC1 molecules were purified by 10% dPAGE. The purified RFD-EC1 was dissolved in water and its concentration of RFD-EC1 was determined spectroscopically. Appropriate amount of ddH_2_O was added to make the final concentration of RFD-EC1 to be 2 µM.

### Preparation of Bacterial Stocks

Culture of bacteria and preparation of their stocks were conducted following our previously established protocol [50]. Briefly, a series of *E. coli* glycerol stocks with defined number of cells were first prepared as follows: A single colony of *E. coli* freshly grown on LB agar plate was taken and used to inoculate 2 mL of Luria Bertani (LB) broth. After shaking at 37°C for 14 h, the bacterial culture was serially diluted in 10-fold interval seven times. The glycerol stocks were then made from the original culture to the final dilution (10^−7^) by mixing glycerol to 15%. It was confirmed by culture plating that the 10^−7^ stock contained an average of 20 cells per 100 µL. Thus, the other 6 stocks contained 200, 2,000, 20,000, 200,000, 2,000,000, 20,000,000 cells per 100 µL, respectively. The stocks of *Bacillus subtilis* were made by the same protocol. All the stocks were stored at −80°C until use.

### Detection limit of RFD-EC1

For this experiment, 500 µL of each of 1 to 10^−7^ *E. coli* stocks prepared above (which translate to 100–10^8^ *E. coli* cells, respectively) were placed in a new 1.5-mL microcentrifuge tube, followed by centrifugation at 10,000 g for 10 min. After the removal of the supernatant, 50 µL of 1× RB containing 2 pmol RFD-EC1 were added to each tube to suspend the cell pellets. Following 1 h reaction, cleavage products were analyzed by 10% dPAGE (Fig. 2A). The detection limit was determined to be the smallest number of cells that caused the RFD-EC1 cleavage.

### Growth Curves of *E. coli* Measured by RFD-EC1 and OD Method

A series of culture tubes were set up that had 1.5 mL of LB, followed by the addition of 500 µL of 10^−7^ *E. coli* stock (which contained 100 cells). These tubes were then placed in a 37°C incubator (with shaking). At 1 h interval 1 ml of the culture was taken out to measure the OD_600_ value. At the same time, 24 µL of the same culture was mixed with 25 µL of 2× RB and 1 µL of 2 µM RFD-EC1. After incubation for 30 min, the DNA in the mixture was recovered by ethanol precipitation. This procedure was repeated 10 times (until 11^th^ h), and the DNA samples were then analyzed by 10% dPAGE (Fig. 2B).

### Monitoring *E. coli* Growth in Microwell Plate by RFD-EC1

Five culture tubes were set up to have 1.95 mL of LB, followed by the addition of 50 µL of 10^−3^ to 10^−7^ *E. coli* stocks (which contained 10^5^ to 10 cells, respectively). These tubes were then placed in a 37°C incubator (with shaking). 50 µL of culture from each well was taken at 0, 1, 4, 8, and 12 h and subjected to ultrasonic treatment to stop cell growth. Thus treated culture was placed in a well of a 96-well plate containing 50 µL of 2× RB with 2 pmol of RFD-EC1. After incubation for 30 min, the fluorescence intensities of the reaction wells were taken using Typhoon 9200, variable mode (GE Healthcare. The data is presented in Fig. 2C.

### Comparison of Detection Sensitivity of RFD-EC1 and OD Methods

A single colony of *E. coli* freshly grown on LB agar plate was taken and used to inoculate 2 mL of LB. After culturing for 14 h with shaking at 37°C, 14 µL of the culture was transferred to 2 mL of LB to do the subculture. After 16 h at 37°C, the bacterial culture was serially diluted in 4-fold interval nine times. The OD value for each dilution recorded (S1A Fig.). Then 1-mL sample of each dilution was placed in a new 1.5-mL microcentrifuge tube, followed by centrifugation at 10,000 g for 10 min. After the removal of the supernatant, 100 µL of 1× RB containing 2 pmol RFD-EC1 were added to each tube and the resuspended cells were transferred to the microwell plate. After incubation for 60 min, the fluorescence intensities of the reaction wells were taken using Typhoon 9200 (S1C Fig.). The cleavage reaction of RFD-EC1 in each well was analysed using 10% dPAGE after ethanol precipitation (S1B Fig.). It was determined by bacterial plating and counting that the 10^−9^ dilution contained an average of 100 cells per 20 µL. Therefore the serially diluted samples should contain 5.0×10^3^, 2.0×10^4^, 8.0×10^4^, 3.2×10^5^, 1.3×10^6^, 5.2×10^6^, 2.1×10^7^, 8.4×10^7^, 3.4×10^8^, and 1.4×10^9^ *E. coli* cells per mL, respectively.

### Inhibition of *E. coli* Growth by Ampicillin Determined by RFD-EC1

A 10 mg/mL ampicillin solution was first made with ddH_2_O. Two 15-mL culture tubes marked “+” and “−” were then prepared; “+” tube contained 1.5 mL of LB with 10 µg/mL ampicillin while the “−” tube contained 1.5 mL of LB without ampicillin. To each tube 500 µL of 10^−7^ *E. coli* cell stock (containing 100 cells) was added, followed by incubation at 37°C (with shaking) for 6 h before 2-min ultrasonic treatment to stop cell growth. Meantime, 90 µL of 1× RB containing 2 pmol RFD-EC1 was transferred into four wells in a black 96-microwell plate with transparent bottom (Corning), which were marked “+”, “−”, “NC” and “Marker”, respectively. 10 µL of cultures from “+” and “−“ tubes were transferred to the “+” and “−” wells. 10 µL of LB were added to the “NC” well while 10 µL of LB with 2 M NaOH was added to the “Maker” well. After 1 h, the plate was scanned for fluorescence intensity (Typhoon 9200) and data is presented in Fig. 3A. The cleavage of RFD-EC1 was analyzed by 10% dPAGE (Fig. 3B).

The effect of ampicillin concentration on *E. coli* growth was tested in similar way by culturing 100 seeding *E. coli* cells in 2 mL of LB containing 1, 2, 4, 8, 16, 32 µg/mL ampicillin for 6 h. The cleavage reaction of RFD-EC1 with the respective culture was carried out in a 96-microwell plate in the same way as described above (Fig. 3C). The cleavage of RFD-EC1 was analyzed by 10% dPAGE (Fig. 3D).

For comparison, the traditional OD method was also used to examine the effect of ampicillin on *E. coli* growth in 2 mL of LB containing 2×10^5^ seeding *E. coli* cells as well as 0.25, 0.5, 1, 2, 4, 8, 16, 32 µg/mL ampicillin (note that 2,000× more seeding *E. coli* cells in the OD method). After incubation for 18 h, the OD value of each culture was measured and plotted as Fig. 3E.

### Fluorescence Assay for Identifying Pore-Forming Molecules

In six microcentrifuge tubes, 150 µL of 10^−3^ *E. coli* cell stock (translating to 3×10^5^ cells) were centrifuged at 8,300 g for 10 min, followed by removal of the supernatant. The cell pellets were resuspended in 50 µL of 1× RB containing 100 µg/mL kanamycin, ampicillin, chloramphenicol, rifampin, trimethoprim or polymyxin B. A positive control was also conducted using 0.2% Triton ×100. The fluorescence signal generation was monitored in each sample in real time using a Cary Eclipse Fluorescence Spectrophotometer. The excitation wavelength was set at 480 nm and the emission was monitored at 520 nm. Readings were taken every minute and the data is shown in Fig. 4A. The cleavage reaction of RFD-EC1 was analysed using 10% dPAGE and the data is provided in Fig. 4B.

### Monitoring Growth Competition between *E. coli* and *B. subtilis* by RFD-EC1

Varying numbers of *E. coli* and *B. subtilis* cells were placed in 200 µL of LB in a 5×7-well plate as indicated in Fig. 5A. The wells in column A were seeded with 10-fold serially diluted (100,000 to 10, from top to bottom) *E. coli* cells only, which served as positive controls; those in column G were seeded with *B. subtilis* cells only also in 10-fold dilutions from top to bottom. The wells in each column indicated as B, C, D, E, F were inoculated with 10, 100, 1,000, 10,000, 100,000 *E. coli* cells, respectively, while those in each row marked as e, d, c, b, a were seeded with 10, 100, 1,000, 10,000, 100,000 *B. subtilis* cells, respectively. After incubation for 18 h at 37°C, the OD_600_ value of each well was measured to assure every well reach the same level (∼0.9). 7 µL of the culture from each well was then mixed with 93 µL of 1× RB containing 2 pmol RFD-EC1. After 1 h, the fluorescence intensity of the reaction plate was obtained using Typhoon and the data is shown as Fig. 5B.

The *E. coli* cell line we used contains a plasmid with an ampicillin resistance gene *while B. subtilis* cell is not resistant to ampicillin. This facilitated the study of whether *E. coli* survived in the well that contained *B. subtilis*. This experiment was performed as follows: after the competition, 10-µL culture from each well was taken to a well in a new 5×7-well plate with 190 µL of LB containing 50 µg/mL ampicillin. The new plate was placed at an incubator. After culturing 37°C for 24 hours, the OD_600_ value of each well was read and the data is shown as Fig. 5C.

## Supporting Information

S1 Fig **Comparison of sensitivity of three detection methods.** (A) OD method. (B) RFD-EC1/dPAGE method. (C) RFD-EC1/plat reading method. RF in C is calculated as (F - F_C_)/(F_max_ - F_C_), where F, F_C_ and F_max_ represent the fluorescence intensity of each well, the control well with no *E. coli* cells, and the well with 1.4×10^9^ *E. coli* cells, respectively.
(DOCX)

S2 Fig **The sequences of synthetic DNA oligonucleotides used in this study.**
(DOCX)
