# Supplementary material for: Examination of Bacterial Inhibition Using a Catalytic DNA
Source: PLoS One. 2014 Dec 22;9(12):e115640. doi: 10.1371/journal.pone.0115640 (PMC4274092; doi:10.1371/journal.pone.0115640)
Supplement: S1 Fig — Comparison of sensitivity of three detection methods. (A) OD method. (B) RFD-EC1/dPAGE method. (C) RFD-EC1/plat reading method. RF in C is calculated as (F - FC)/(Fmax - FC), where F, FC and Fmax represent the fluorescence intensity of each well, the control well with no E. coli cells, and the well with 1.4×10^9^ E. coli cells, respectively. (DOCX) [file pone.0115640.s001.docx]

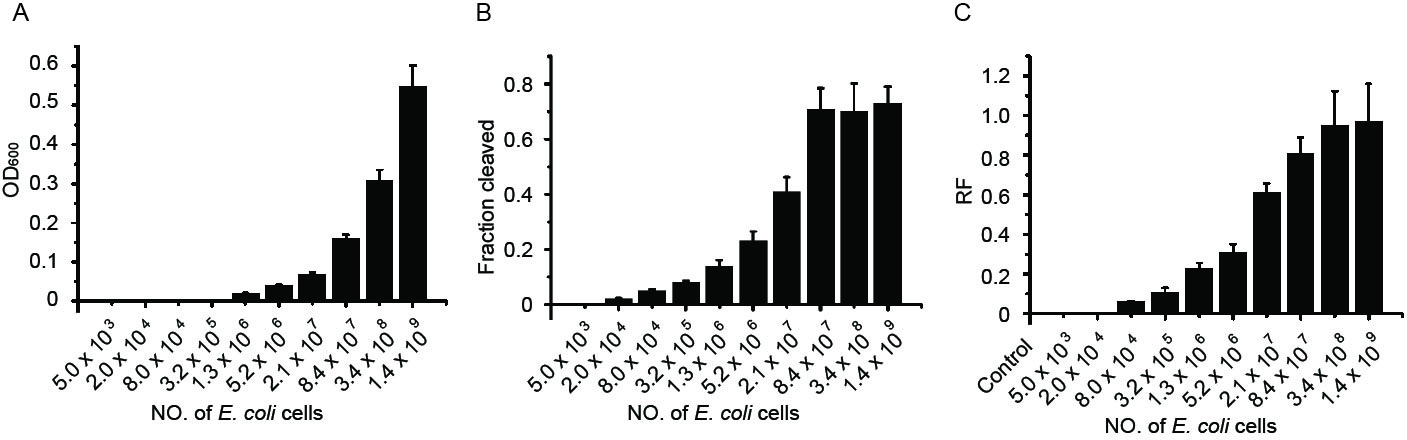


**Figure S1**. **Comparison of sensitivity of three detection methods.** (A) OD method. (B) RFD-EC1/dPAGE method. (C) RFD-EC1/plate reading method. RF in C is calculated as (F - F_C_) / (F_max_ - F_C_), where F, F_C_ and F_max_ represent the fluorescence intensity of each well, the control well with no *E. coli* cells, and the well with 1.4 × 10^9^ *E. coli* cells, respectively.
