# Supplementary figures and images for: Examination of Bacterial Inhibition Using a Catalytic DNA
Source: PLoS One. 2014 Dec 22;9(12):e115640. doi: 10.1371/journal.pone.0115640 (PMC4274092; doi:10.1371/journal.pone.0115640)

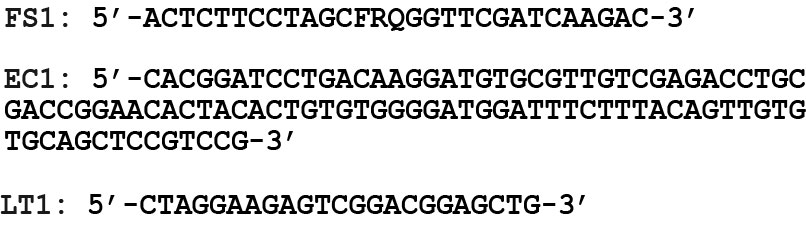


**Figure S2.** The sequences of synthetic DNA oligonucleotides used in this study.

Supplement: S2 Fig — The sequences of synthetic DNA oligonucleotides used in this study. (DOCX) [file pone.0115640.s002.docx]
